# A holistic framework for assessing the uptake potential of EU-funded security research and innovation project results

**DOI:** 10.12688/openreseurope.19711.3

**Published:** 2026-04-29

**Authors:** Marcel van der Lee, Clara Peters, Marcel van Berlo, Luis Unzueta, David Ríos, Sirra Toivonen, Gonçalo Cadete, Björn Hoog, Salvatore Vicari, Ernesto La Mattina, Laurynas Adomaitis, Alexei Grinbaum, Hassane Essafi, Souzanna Sofou, Katerina Valouma, Ilias Gkotsis, Nikos Chantavas, Luke Bates, Helen Gibson, Babak Akhgar, Christelle Magimel, Robert Kuch Wesolowski, Anders Åström, Zakarias Subeh, Eleni Darra, Michalis Angelou, Dimitrios Kavallieros, Nicholas Vretos, Theodora Tsikrika, Stefanos Vrochidis

**Affiliations:** 1 TNO, Nederlandse Organisatie Voor Toegepast Natuurwetenschappelijk Onderzoek, Den Haag, The Netherlands; 2 Fundación Vicomtech, Basque Research and Technology Alliance, Donostia-San Sebastián, 20009, Spain; 3 VTT, Technical Research Centre of Finland, Espoo, Finland; 4 INOV - Instituto de Engenharia de Sistemas e Computadores Inovação, Lisboa, Portugal; 5 Fraunhofer Institute for Technological Trend Analysis (INT), Euskirchen, Germany; 6 Engineering Ingegneria Informatica SPA, Roma, Italy; 7 Commissariat a L’Energie Atomique et aux Energies Alternatives,, Paris, France; 8 Satways Ltd, Integrated Security and Defense Solutions, Private Security Services Company, Athens, Greece; 9 CENTRIC (Centre of Excellence in Terrorism, Resilience, Intelligence, and Organized Crime Research), Sheffield Hallam University, Sheffield, UK; 10 SDIS 78, Yvelines Fire and Rescue Service, Versailles, France; 11 Polismyndigheten Swedish Police Authority, Stockholm, Sweden; 12 Information Technologies Institute, Centre for Research and Technology-Hellas, Thessaloniki, Central Macedonia, Greece

**Keywords:** MultiRATE, TRL, Holistic Readiness Level, Maturity Assessment, Research and Innovation, EU Civil Security Domain

## Abstract

The Technology Readiness Level (TRL) has been adopted since 2014 within the European Union (EU) as a metric to evaluate the maturity of results from EU-funded research and innovation projects. This metric is crucial for distinguishing between innovation actions aimed at early-stage innovations and market-ready solutions. Ideally, EU-funded research and innovation projects should lead to the development of innovative concepts and technologies by EU industries, which in turn enhance security capabilities within EU member states. However, there is a notable challenge: the adoption rate of outcomes from EU-funded security research and innovation projects is not as high as expected. The current TRL maturity assessment method is insufficient in exposing the possible cause of the limited uptake by fully pointing out where the development is lacking. The TRL’s limitations include a lack of comprehensive assessment from various perspectives especially in the civil security research and projects, which is necessary to bridge the gap, often referred to as the “valley of death,” between project results and their effective adoption. To address these shortcomings, in the MultiRATE EU research project we propose a holistic framework that enhances the TRL scale by adding additional Readiness Levels (RLs) for a more complete evaluation of security projects. These include the Societal RL (SocRL), Security RL (SecRL), Legal, Privacy and Ethical RL (LPERL), Integration RL (IRL), Commercialisation RL (CRL), and Manufacturing RL (MRL). In this open letter, we explain the background of the design considerations of this framework. Our goal is to define and integrate these seven Readiness Level (RL) dimensions and an investment forecasting tool to support policy makers, practitioners, and investors in bridging the “valley of death” between research and adoption.

## Plain Language Summary

The Technology Readiness Level (TRL) is a tool used in the EU to measure how mature new technologies are, especially those funded by the EU. It's important for figuring out if these technologies are ready for the market. However, many EU-funded security projects aren't being adopted as much as expected. The current TRL method doesn't fully explain why this is happening because it doesn't look at all the necessary angles.

To fix this, the MultiRATE EU project suggests a new framework that adds extra levels to the TRL. These new levels include societal impact, security, legal and ethical considerations, integration, commercialization, and manufacturing readiness. This more detailed approach aims to better evaluate security projects and help them get adopted more effectively. The white paper explains how this new framework can be designed and implemented to improve the adoption of these projects.

## Introduction

In the 1970s, the National Aeronautics and Space Administration (NASA) developed new methods for evaluating the maturity of technologies and the associated risks of technology development. ^1^ Initially, a seven-level Technology Readiness Level (TRL) scale was introduced to determine whether a technology was sufficiently mature for deployment in space. This TRL scale has since been refined into the current nine-level TRL scale. ^2, 3^ This standardized method is widely used by both the public and private sectors. In 2010, the European Commission recommended the use of the TRL scale for EU-funded research and development projects [1]. As a result, the research and development community adopted this scale in 2014 as part of the EU Horizon 2020 program for assessing the development potential and results of the projects. ^4, 5^

The TRL concept is relevant for Horizon Europe’s Pillars 2 and 3. Pillar 2 distinguishes between Research and Innovation Actions (RIA) with lower maturity/TRL (4–5) and Innovation Actions with higher maturity/TRL (6–7) requirements. ^2^ In the European Innovation Council (EIC) programs in Horizon Europe’s Pillar 3, focused on innovation, TRL requirements are included in the different calls for the EIC grants. ^4^ The EIC programs distinguish between three grants, depending on the TRL:

• **Pathfinder:** For early-stage technological development in TRL 1 to 4, grant aiming to provide support to further research and develop of emerging breakthrough technologies.
• **Transition:** Assists technologies in phases beyond proof of principle at TRL 3 or 4 to develop and validate their feasibility towards an outcome TRL of 5–6.
• **Accelerator:** Aids innovations at a TRL 5–6 up to 8, helping scale-up and introduce the innovation to the market.

TRL is used in EU-funded research and innovation projects to distinguish maturity levels and select appropriate grants for concept and product development. Further applications of TRL within the context of EU-funded research projects include the assessment of the maturity of the output of projects, and the maturity growth of systems and solutions during the research projects execution.

A TRL-9 product is expected to be a mature innovation ready for industry and end-users. However, even after reaching TRL-9, there is no guarantee that an innovation or solution will be adopted by the industry for production or acquired by end users. Especially for the EU civil security research and innovation projects, a key challenge remains in improving the uptake of innovations. The factors influencing this uptake have been analyzed in a relevant study by DG HOME, ^6^ and are further discussed in a proceeding chapter of this work.

**Figure 1.**
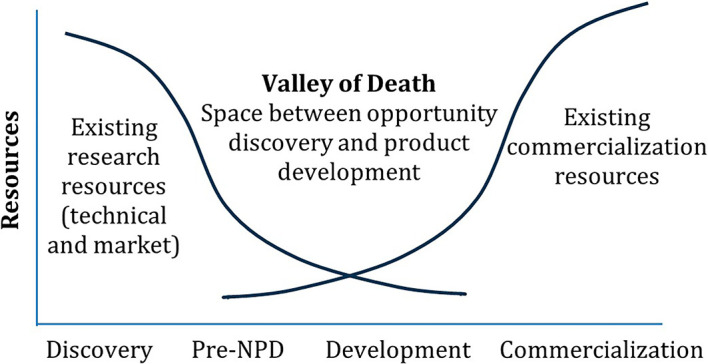
The Valley of Death. ^7, 8^ Pre-NPD (New Product Development) involves the activities and decisions made after initial R&D but before full-scale product development begins. (Figure © 2010 Product Development & Management Association. Figure reproduced with permission from John Wiley and Sons, figure adapted from 11).

The key challenges are usually associated with factors such as market fragmentation, along with other demand-side, supply side, institutional barriers identified in the literature, etc. These challenges are often faced by EU security research and innovation projects in their effort to bridge the gap between project outcomes and reaching the maturity level needed for smooth industry and/or end-users’ uptake. This gap is commonly referred to as the “valley of death”, ^7, 8^ a metaphor that highlights, among others, the difficulties in securing funding and support during the transition period ( Figure 1). As a medium to support the bridging of the valley of death, relevant work has suggested a holistic approach to Innovation Management ^9, 10^ that is applied in this work.

In advancing technological innovation, a Holistic Readiness Level (HRL) calculator integrating multiple RL dimensions like the one proposed in MultiRATE can provide a structured way to evaluate specific aspects of readiness, offering granular insights into progress and feasibility.

This open letter is meant to describe at a conceptual level how a successor of the TRL metric could look like. We will not present empirical outcomes nor finalised metrics. In this open letter we will take a closer look at the background of the design considerations of the MultiRATE framework which are merely the factors that contribute to success and failure of the uptake of EU-funded security cluster 3 program projects (Civil Security for Society), the limitations of TRL and the MultiRATE approach to overcome all these challenges. Specifically, we will explain at a conceptual level the considered seven RL dimensions (TRL, SocRL, SecRL, LPERL, IRL, CRL, MRL) and the design factors to build the holistic framework. We will discuss insights on its implementation considering that each RL scale comprises a set of indicators and methodologies that must be integrated and aligned for an effective holistic maturity assessment, considering the specific characteristics of each RL dimension. Moreover, an investment forecasting tool could help with the management of security research projects and their resources by relating RL improvement and the required effort for that. Given the widespread use of TRL in recent decades, MultiRATE proposes a TRL investment forecasting tool with a methodology that can extend to other RL dimensions as more data becomes available.

## Methodology

The factors that contribute to success and failure of the uptake of EU-funded security cluster 3 program projects (Civil Security for Society) and the limitations of TRL are collected via a narrative literature review that was started with a search on main keyword (“TRL shortcomings”, “Factors uptake EU Research”, “Evaluation EU funded research”) followed by citation research (snowballing approach).

For the purpose of assessing the maturity of EU R&D project outputs in the security domain, seven existing readiness levels (RL) were studied, assessed, and modified. The keyword-based searches relevant to each RL were done across major scientific databases including also European Commission reports and policy documents, while previous work on the subject conducted by the consortium partners was included. For this literature review, no strict inclusion or exclusion criteria were applied. The collected literature was synthesized qualitatively to identify gaps and limitations in existing assessment approaches.

The selection, assessment, and modification of the seven Readiness Level (RL) dimensions were informed by this literature review, as well as by the work conducted in the frame of the MultiRATE project, which focused on analyzing cross-disciplinary readiness level methodologies, identifying stakeholder requirements, and defining the structure and scope of the proposed RL framework.

## Factors that limit uptake of EU research and innovation project results in the security domain

The EU-funded security cluster 3 program addresses persistent security threats, including cybercrime, and natural or man-made disasters. ^11^ According to the Horizon Europe Strategic Plan, the research and innovation actions in this cluster should contribute to (1) reduce losses from disasters, (2) facilitate travel for legitimate passengers and shipments into the EU while preventing travel for non-legitimate ones, (3) tackle crime and terrorism more effectively and (4) increase cybersecurity and create more secure online environment. ^11^

From evaluations of EU funded programs (7th EU Framework, Horizon 2020), ^12, 13^ and from evaluations focused on the security cluster ^14– 16^ (PASR, Horizon 2020, Commission Staff Working Document Study on the Factors Influencing the Uptake of EU-Funded Security Research Outcomes) and scientific literature on the impact of EU funded research, several factors emerge that contribute to hindering the uptake of EU-funded security project results ^17– 19^:

• **Market Fragmentation:** Administrative responsibilities, legal frameworks and operational practices, as well as security challenges, differ considerably among member states, which complicates the development of universally applicable tools. In the crime and terrorism subdomains in particular, differences in national legislations are an obstacle to uptake, as solutions must be tailored to local requirements. This market fragmentation hinders the wider adoption of successful research project solutions across the EU. ^6, 20^
• **Quality of Information flows:** The quality and quantity of information sharing on EU-funded security research innovations and results is often inadequate. Additionally, a key barrier to uptake involves the sensitive nature of certain security domains, making it challenging to widely disseminate research outcomes. ^6, 20^
• **Insufficient output maturity for uptake:** EU funded research project results often do not reach the level of development required for commercialisation by the end of the project. This leads to a need to find funding for follow-up development while simultaneously it is too early for end-users to assess the take-up of new technologies, systems, approaches and knowledge. ^6, 14, 15, 20^
• **Lack of foresight and evolving end user requirements:** The lack of long-term planning across multiple security innovation domains may be a barrier to uptake.
• **Protection and clarity of IP rights:** IP rights protection and clarity can be a barrier due to restrictions on transferring IP between projects. Both academic literature and EC studies have similarly suggested that IP rights can act both as a barrier and facilitator for uptake.
• **Challenges associated with public acceptance:** Uptake also implies the involvement of various stakeholders, including direct involvement of practitioners and industry, but also indirect recognition of the policy sector for support and buy-in.
• **Restrictions of an institutional market:** The security market is one of the few markets in which public sector authorities represent the primary (and sometimes only legal) customers for solutions and technologies, creating unique challenges for uptake. This factor, in conjunction with a complicated regulatory framework, makes adaption hard to predict due to non-standard market dynamics and limited market visibility.

In addition to these factors, there may be issues related to the metrics used (mostly TRL) to measure the maturity of innovations and track their progress. Inappropriate metrics may lead to imbalances in critical aspects necessary for successfully navigating the R&D “valley of death”. Therefore, it is important to examine the limitations of TRL.

## TRL limitations

Although the TRL scale is used widely and has only marginally changed since its inception, several shortcomings have been identified over the years. First, there is a lack of precise definition of the individual levels. No sound definition of the individual levels has yet been fully explained and exemplified, and a succinct definition of terminology is lacking. ^1, 21^ Olechowski *et al.* ^3^ found issues with the subjectivity of the TRL assessment and imprecision of the scale. Problems might emerge when RLs proliferate and are used without a commonly agreed definition or when they are implemented without the support of adequate tools and methods to carry out a reliable assessment. A second issue is that technology hand-off is not considered. One of the main goals of the original NASA TRL method, was to use the assessment to get insight in the right moment for transferring technology between departments within NASA to advance its development efficiently. This Technology hand-off from one party to another needs to be done at certain specific TRL levels. Different types of organisations are needed at different TRL levels. The EC TRL version does not address this problem, although the EU has established different fund types for research and development at different TRL levels. ^22^ Another issue with TRL is that TRL level differs from the context of the foreseen application environment. A technology maturity level needs to be considered within the context of the foreseen application. When different applications are foreseen, a technology has multiple TRL levels concurrently. ^22^ Changes in the application environment should lead to guidance on the change of the TRL. ^3^

TRL does not include any information on the possibility and difficulty of further developing a technology to a higher level, which would be useful to get insight into the risks of that development. ^21^ It is now an assessment at one point of time, and it does not give insight into the needed effort or technological challenges to bring the TRL to a higher level. ^3^ To solve this issue, the founder of the TRL scale, Mankins, proposed to use the notion of “R&D degree of difficulty”. ^23^ Other aspects are the necessary costs to step to a next level. The costs to step from one TRL level to another increase with the maturity level. 90% of the costs will be spent to come from TRL 7 to TRL 9. ^22, 24^ Costs are multiplied when transitioning from TRL 5 to TRL 6 and then again to TRL 7. ^22^ Another issue is that the TRL scale is often used for multiple purposes, which might need different assessment aspects. The TRL scale can have multiple purposes, like communication, providing support to project planning or aiding investment decisions. ^1^ For example, EU’s High-Level Group on Key enabling Technologies (HLG-KET) recommended using TRL as a tool for assessing the results and expectation of the projects. The question is if the TRL scale needs to be adjusted for each specific purpose. For example, assessing eligibility to access specific funding. For this purpose, in the Horizon 2020 program, additionally to a TRL assessment it also asked for mid to high TRL programs to provide a business plan for future development. ^1^

The TRL scale originates from NASA in the 60s/70s of the previous century, when software did not play such a dominant role within innovations as today. At the time that the TRLs were conceived at NASA, hardware was emphasised significantly more than software. ^21, 25^ The US-GAO argues that evaluation of software is more challenging than evaluating hardware because it lacks physical properties that can easily be characterised, measured and tested. ^26^ Apart from traditional software applications, with the advent of machine learning applications, the traditional TRL levels may no longer apply.

For the assessment of the maturity of an integrated system consisting of various components and/or technologies, each with its own maturity level, the TRL method is less suitable. Although the higher TRLs mention the notion of system maturity, implying a composition of multiple components and/or technologies, still the levels offer limited insight into integration, which is a key challenge faced by development programs. The aspect of research solutions that will need various technologies is not addressed. ^1^ Olechowski *et al.* ^3^ found that integration and connectivity was found to be the most critical challenge overall in applying the TRL levels. System architecture connects different components within a system through interfaces. The maturity of these interfaces could be improved by coming up with new ways of connecting components. This interface maturity is not covered in the TRL framework. ^3^ This also raises the question as to which components in a system need to be assessed to get an assessment of the overall system. Is it necessary to assess all the components? Or is it sufficient to restrict assessment to the components of high technology risk? And what happens if one component is replaced in a system? Is it necessary to re-assess all components? Or only the new component? And what if the new component has a lower TRL than the original one. Does the entire system then become that lower TRL?” ^3^

The TRL scale is linear and does not consider the cyclical, iterative, or non-monotonic nature of technology development. ^27^ And how to handle step backs in development? EARTO mentions that design flaws that emerge during initial manufacturing can throw back a technology to earlier stages of development, for instance requiring more R&D to achieve technological feasibility. ^1^ Moreover, TRL-9 does not mean ready-for-the-market. For example, commercialisation RLs are at a rather low level when TRL is 9. ^22, 28^ In order to assess if a technology is ready-for-the-market, aspects like manufacturability and readiness of manufacturing technologies have to be taken into account as well. Furthermore the readiness of an organisation to implement the innovation, ^1^ as well as the ethical and societal aspects ensure alignment of the technology with the existing ethical and societal norms. ^29^

## The MultiRATE approach

In the past years, the European research community has identified that assessing correctly the RL is crucial as it enables a clear understanding of the technological maturity and exploitation feasibility of research outcomes. Furthermore, it aids in informed decision-making, effective resource allocation, and timely identification of promising technologies for further development and implementation. In this context, the ambition of the MultiRATE EU research project ^30^ is to develop a holistic, homogeneous, and harmonized RL evaluation methodology and calculator for R&D projects and solutions in the security domain, which will be made available to the EU R&D community. For an improved maturity assessment method, from the above-described factors that currently hinder the uptake of EU project and from the shortcomings of the current TRL method, we have derived eleven design considerations for MultiRATE:

1. **Define RLs with clarity and precision:** Ensure that the method includes a comprehensive and precise definition of each RL. This involves developing clear terminology and structured descriptions for all RL stages to address the ambiguity currently observed in RL assessment methodologies.
2. **Incorporate application environment as a factor:** Recognize that the maturity of a product can vary depending on its application environment. Include factors such as language barriers, organizational contexts, and procedural compatibility to reflect the variability in RLs across different scenarios and stakeholders.
3. **Customize the RL method for its intended purpose:** Tailor the method to specific objectives, such as planning, investment decision-making, or progress tracking. Ensure that the level of detail matches user needs—neither overly simplistic nor excessively complex—to maximize usability and relevance.
4. **Account for organizational hand-offs in the RL assessment:** Integrate checks to ensure that the right type of organization or department is involved at each RL. Addressing the technology hand-off process is critical for smooth transitions between development stages.
5. **Include interoperability commitment as a criterion:** Incorporate a factor to assess the commitment of stakeholders to harmonize technologies, processes, and procedures. This is crucial to address barriers that limit the widespread adoption of research outcomes, especially in collaborative or multi-stakeholder environments.
6. **Evaluate how well the solution meets end-user needs:** Assess the extent to which the product satisfies the requirements and expectations of its end-users. Products that fail to meet these needs cannot be considered mature, and this evaluation should include an analysis of funding availability to measure added value for end-users.
7. **Factor in trust, knowledge, and engagement of end-users:** Include criteria to evaluate the level of trust, awareness, and involvement of end-users and stakeholders. These elements are essential to fostering acceptance and uptake of the product.
8. **Assess costs and risks of advancing RL levels:** Provide insights into the expected R&D efforts, costs, and risks associated with advancing the maturity level of the product. This information supports informed decision-making regarding further development.
9. **Consider financial and procurement factors:** Incorporate the availability of financial resources and the presence of pre-procurement or procurement projects into the method. Address how these factors influence the demand for and development of security products.
10. **Include regulatory context as a driver:** Factor in the presence, absence, or evolution of relevant regulations that drive the need for the product. Harmonized regulations across regions can significantly enhance product adoption.
11. **Address licensing and IP rights:** Assess the level of agreement or effort required to resolve licensing and intellectual property (IP) rights issues. These aspects can present significant barriers to the uptake of research outcomes and must be proactively managed.

**Figure 2.**
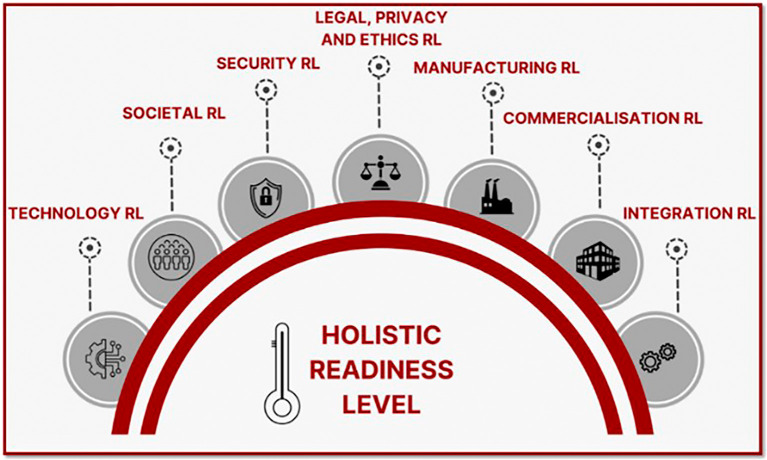
MultiRATE’s RL dimensions.

Following all these considerations, MultiRATE proposes seven RL dimension scales, which can be used individually or combined into a holistic RL scale ( Figure 2), and an investment forecasting module to estimate the costs needed to increase RL maturity. The MultiRATE framework is designed to support a broad range of stakeholder groups across the EU civil security research and innovation ecosystem. While the holistic assessment can be applied at policy level, the individual RL dimensions are intended to serve different primary user groups depending on the maturity aspect being evaluated. TRL and MRL are particularly relevant for R&D, industry partners, and investors seeking to understand technological maturity and manufacturing feasibility. SocRL and LPERL target policy makers, societal actors, and ethics/legal experts, helping identify societal acceptance, legal compliance, and privacy implications. SecRL and IRL are highly useful for practitioners and system integrators assessing security performance and integration readiness. CRL directly supports commercialisation teams, investors, and exploitation managers evaluating market readiness. At the same time, the Holistic RL (HRL) is intended to provide a cross-dimensional view that can inform policy-level evaluations, strategic investment decisions, and diagnostic analyzes of systemic barriers to uptake.

Next, we introduce each of these artefacts and our approach to designing and validating them.

### Technology RL (TRL)

The TRL calculator developed within the scope of MultiRATE project aims to provide the European R&D community and relevant European Commission Agencies with a methodology for measuring how mature a particular technology is in EU R&D project solutions. In MultiRATE, the TRL will keep the widely used 9 TRL levels and the definitions already used in EU R&D projects and will focus the efforts on developing appropriate indicators per level for assessing a technology’s stage of development while taking various aspects like research, experimentation, and testing into account. Thus, TRLs will increase when a technology develops from early idea phases to fully operational solutions, demonstrating the technology’s rising level of maturity and declining any foreseen risks. The TRL framework developed by MultiRATE also incorporates software and AI development by emphasizing the evaluation of intangible assets and their integration within complex systems, ensuring a comprehensive and adaptable readiness level assessment. TRL framework will enable stakeholders to learn more about the readiness of the technology for deployment as well as practical application of hardware and/or software solutions. MultiRATE’s TRL evaluation framework will comprise four distinct indicator categories: Technology Preparation & Requirements, Documentation, Operability & Continuity, and Evaluation & Usability. These categories serve as key aspects for assessing the readiness and effectiveness of an element.

### Societal RL (SocRL)

The SocRL calculator assesses the level of adaptation of an innovation (e.g., the use of a new piece of equipment, system, software, methodology, or procedure within a context it has not been used before) to be successfully adopted by society. Previous work has been carried out into societal readiness, with the most well-known scale coming from Innovation Fund Denmark. ^31^ However, the existing works on societal readiness are fragmented and incomplete for the purposes of MultiRATE’s proposed comprehensive assessment framework; additionally, past research tends to focus on the areas of energy, decarbonisation, and sustainability, ^32, 33^ and therefore more work is needed to apply these societal considerations to the security domain, where they are just as, if not more, relevant than ever. The MultiRATE SocRL will include measures such as the take-up and acceptance of an innovation by society. It will feature levels ranging from the initial identification of the societal need, societal good, and associated readiness aspects of the innovation in question, all the way up to finally proving its benefit within society after launch on the market. It predicts the readiness of and helps prepare an innovation along its journey of adoption.

### Security RL (SecRL)

There only exist a few security maturity models that focus on the evaluation of an innovation, product or solution directly. However, these models are usually designed only to assess a particular type of technology and are not well-established and mature. Such models are Microsoft’s Security Development Lifecycle for securing software, the Cloud Control Matrix, Pirinen’s Common Information System Maturity Validation Resilience Readiness Level (CISMV-ResRLs) or Straub’s Cyber Capability Readiness Level (CCRL) system. ^34– 37^ But while looking closer, the CCRL is rather designed as a TRL-scale tailored to systems with a cybersecurity purpose alone or the cybersecurity tool or measures perse.

Considering the information mentioned above, we can argue that tools aligned with MultiRATE’s purpose for evaluating the security readiness or maturity of a general innovation or solution, meaning technology or a process, do not exist. Hence, such a tool will be developed inside the MultiRATE project. ^38^

MultiRATE’s SecRL calculator will allow users to assess the security level of a specific element (e.g., product, system, or process) and its assets, considering the threat environment and implemented security measures. Designed as a progressive framework, the calculator will evaluate indicators for each level, ranging from security consideration aspects to operational security validation aspects. It will determine the achieved security level, calculating fulfilment percentages, and highlighting areas for improvement, providing guidance on further steps to enhance security readiness. This approach will provide users with a comprehensive view of the overall security posture, facilitating informed decision-making and targeted strategies to effectively mitigate risks faced by the element in its operational environment.

### Legal, Privacy and Ethical RL (LPERL)

Currently, no unified (Legal, Privacy and Ethical RL) LPERL exist for security or generalist technology or applications. ^38^ This is the biggest gap in the analysis, meaning that the methodology, the indicators, and their weights will need to be developed in the scope of MultiRATE. Literature review shows several levels of non-security specific and non-unified levels that can be relevant for LPERLs. For example, there are legal readiness levels specific to machine learning ^39^ and blockchain technologies, ^40^ however they do not address other concerns or the security field. One machine learning assessment tool considers ethical evaluation as part of the TRL structure, which can be used for structural analogy. ^29^ There are no accepted frameworks for ethical or privacy readiness levels, however ethical readiness evaluation tools are developed by other projects in the cluster, like TechEthos (generalist) ^41^ and STARLIGHT (security specific). ^42^

MultiRATE’s LPERL is a specialised metric designed to evaluate the alignment of a solution with legal standards, privacy norms, and ethical principles. By addressing these related challenges proactively, the LPERL aims to ensure that legal and ethical issues are adequately addressed during the development phases. Therefore, the intended benefit of the LPERL calculator developed in MultiRATE is to determine the degree of alignment with European values and rights, provide guidance in LPE (Legal, Privacy and ethics) by design and promote responsible application of solutions. More specifically, the purpose of the LPERL is to iteratively indicate (i.e. the tool should be used multiple times during development and results should be compared to each other) the ethical, privacy, and legal readiness of products/outcomes, and raise awareness of potential current and downstream issues in the evaluated aspects. It will be developed as a modular questionnaire that is customized based on the features of the evaluated product/outcome. Modules will consist of a general ethical module, law enforcement module, personal data module (for non-law enforcement applications), and an AI module. Each module will consist of indicator questions that address ethical, privacy, and legal aspects for different stakeholders and types of technologies.

### Integration RL (IRL)

The IRL is a metric for assessing the maturity of an element (product, system, process) to be effectively integrated into a larger or operational environment. This assessment framework is designed to systematically examine the interactions and dependencies between various integration points, helping stakeholders understand the current level of readiness for seamless incorporation. One of the key benefits of the IRL is its role in identifying potential risks and development areas requiring further engineering work, thereby reducing the likelihood of complications when integrating sub-elements into a broader system. By doing so, it not only addresses immediate technical issues but also highlights areas that need attention to meet system requirements. This allows for more informed decisions on whether to integrate certain technologies, especially in avoiding the inclusion of outdated systems, or those that may not yet be fully mature. Additionally, it assists stakeholders in determining the standards, documentation, and interoperability needed for a successful integration, guaranteeing that technology capabilities match more general system objectives. Overall, the IRL offers a structured approach to mitigate risks and guide the maturation process of components and systems for smoother integration into complex environments. Although there are existing works in the literature that establish scales for assessing the level of integration readiness, e.g., 43– 45 among which one of the most notable contributions is the approach presented by Sauser et al., ^46^ they are generally designed with a specific objective in mind. As a result, they often lack the comprehensiveness required to support the holistic assessment framework proposed by MultiRATE. This limitation highlights the need for a more comprehensive and adaptable approach to effectively address the diverse requirements of integration readiness.

### Commercialisation RL (CRL)

Based on earlier work on CRLs, MultiRATE’s enhanced CRL will be adapted to follow the Holistic Innovation Management methodology. Utilising this methodology ^9, 10^ the proposed combined strategy will be followed for Intellectual Property management, data management, dissemination, communication and exploitation throughout the lifecycle. This will be realised with a multi-level assessment process. For each of levels, indicators will be offered, and their evaluation will determine the readiness for commercialisation of the element under study. The indicators’ types include various aspects, including Intellectual Property Management, Market & Competition Landscape, Team/Consortium Expertise, Solution Definition/Design/Development (including required certifications and regulatory requirements), Exploitation Plan, Manufacturing/Supply chain (including end-user engagement). Overall, as explained in 10 the enhanced CRL aims to support an exploitation team in assessing their product’s readiness for the market, while its indicators will also serve as guidelines to enrich the action plan in every level, improve the product’s commercialisation strategy, and consequently, the innovation adoption. By overcoming all barriers, the element will gradually reach the final CRL level and will therefore be ready for commercialisation.

### Manufacturing RL (MRL)

The goal of the MRL is to support industrialisation managers and R&D teams in managing manufacturing risk and ensuring the manufacturability of products in the transition from R&D to production. Potential investors of a new product can also benefit from having an accurate assessment of product’s MRL. The MRL is a measure to assess the maturity of a given product/system/process from a manufacturing perspective. In more detail, MRL offers decision makers a common framework to assess the progress and the risks that are associated with the manufacturing of an element under development. Besides defining the level of manufacturing readiness, MRL aims to highlight manufacturing, financial and operational gaps, and set the baseline for more efficient risk management and manufacturing feasibility. By embedding the MRL within the broader MultiRATE readiness framework, the project will ensure that manufacturing considerations are comprehensively evaluated alongside other critical domains, enabling a seamless transition from R&D to production while supporting risk mitigation and informed decision-making across all RLs. The MRL framework within MultiRATE will be based on three MRL calculators i.e. DoD, ^47^ AFRL ^48^ and DHS ^49^ and will use various indicator types, including Manufacturing process, Supply chain readiness, Production capacity, Quality control and assurance, Manufacturing equipment and machinery, Cost and efficiency, Training and workforce, and Regulatory & compliance.

### Holistic RL (HRL)

Using the individual RL dimensions, like those presented above, or any other available in the related literature, apart from TRL, can give us a better understanding of a system’s overall maturity than using TRL alone. However, these RL dimensions can be quite heterogeneous (i.e., different number of levels, different relations between indicators and levels, etc.) and their alignment might not be clear enough (e.g., interrelations among indicators, groups of indicators, levels, etc.) without a proper harmonization among them. This can add difficulties for a proper holistic maturity assessment. Current state-of-the-art methods for assessing the maturity of concepts like systems, technologies, and organizations use a holistic approach by integrating different maturity scales. Some of these methods adopt the same number of levels for each RL scale, ^27, 50^ while others do not. ^51– 53^ The former require a design of the indicators that allows aligning directly the levels of each RL scale (i.e., a 1–1 same level mapping), while the latter align the levels of different RL scales from a higher-level perspective to assess the holistic maturity. Thus, the latter allow having RL scales with different number of levels. This higher flexibility is more convenient for MultiRATE as the considered RL scales are quite heterogeneous. For this purpose, MultiRATE’s holistic RL (HRL) assessment acts as a common ground for their integration, considering all their specific characteristics. Our goal in its design is to incorporate all RL scale indicators and methodologies but also preserving the unique aspects of each RL dimension. This means that although we might consider some modifications in the RL scales to harmonize them from the holistic point of view, we should also keep their standalone usefulness. For instance, the levels of this HRL could be aligned to some indicators, group of indicators, levels of each RL dimension and, at the same time, to some specific events related to holistic maturity progress (e.g., having accomplished some technological, societal, legal, commercial, etc. achievement). Once the RL dimensions alignment is achieved with the support of this HRL, the holistic assessment score obtained by this HRL can also give us a quick understanding of the achieved holistic maturity that can be complemented with a more detailed review of the scores obtained in each RL dimension.

### Forecasting module

MultiRATE’s forecasting module aims to predict how much funding is needed to increase an element’s RL or how many levels RL will rise using a given budget. Research person months (PMs) will be used over money as a measure, for accuracy and comparability, as they reflect human effort overcoming the differences between EU states in wages or other costs. Public datasets will be utilised to train the model effectively, before using it on the gathered relevant data. Challenges to be overcome include varying data recording across organisations, introducing subjectivity, and standardisation difficulties. The user will be able to input features related to effort and development duration and distribution, receiving a visual 3D representation of predictions based on PMs, time, and RL progression.

### Development and validation methodology

**Figure 3.**
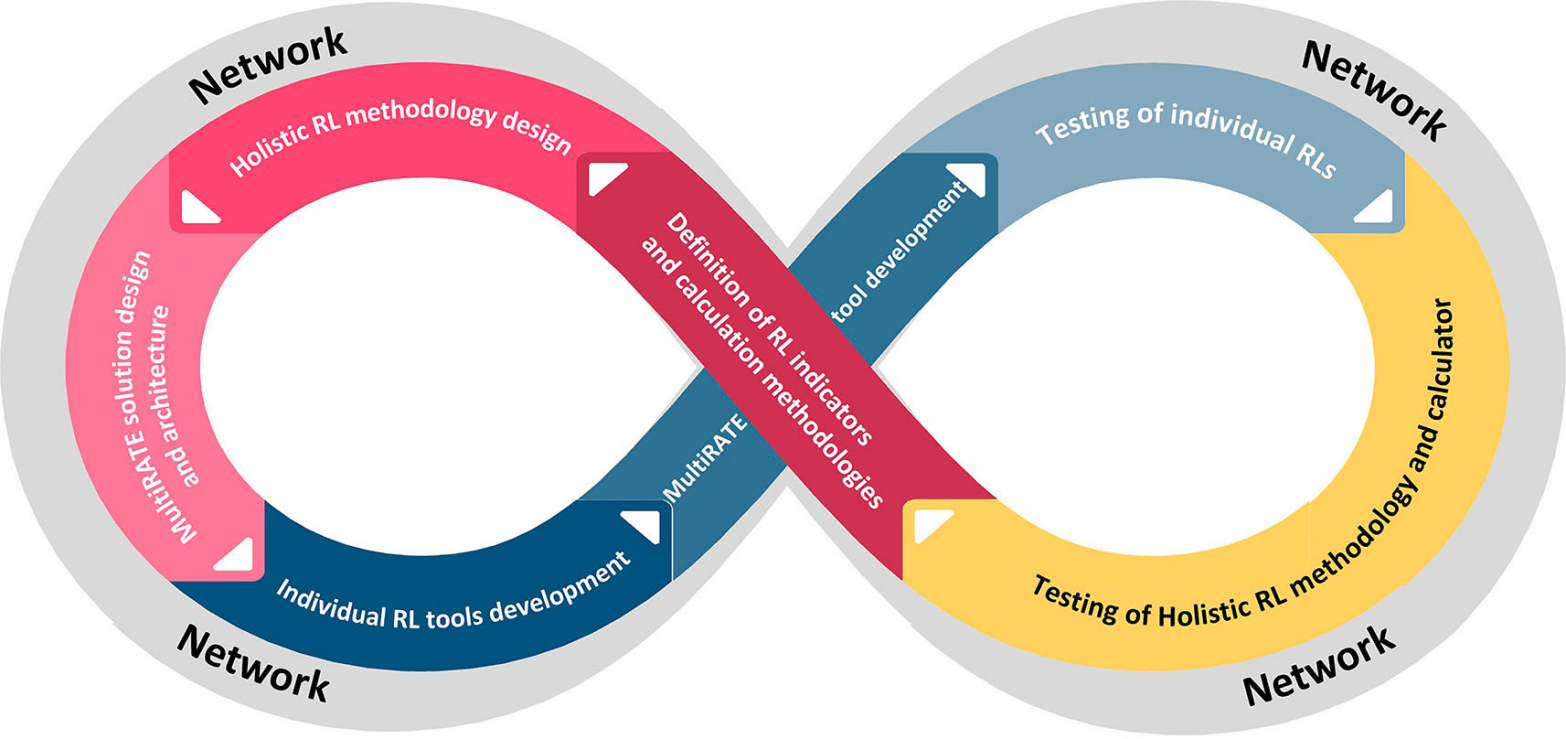
Overall MultiRATE development methodology.

The overall development methodology of MultiRATE will be based on continuous development, testing and updating cycles. The establishment of a network of collaborators for the project will serve as the cycle’s initial starting point. This network will be composed of the project’s partners, networks to which the partners belong, as well as specific businesses from the commercial, public, and research sectors, covering the whole EU R&D community. The network participants will be the end-users of the project, providing significant input regarding the requirements, the indicators, and the validation process. Throughout the cycles, it will operate continuously ^54^ ( Figure 3).

**Figure 4.**
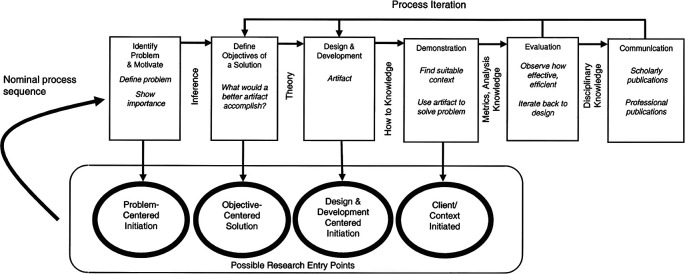
DSRM process model, taken from 58. (Figure © 2008 M.E. Sharpe, Inc from K. Peffers, T. Tuunanen, M. Rothenberger and S. Chatterjee, “A Design Science Research Methodology for Information Systems Research.” Journal of Management Information Systems, vol. 24, no. 3, pp. 45–77, 2008. Figure reprinted by permission of the publisher (Taylor and Francis Ltd., http://www.tandfonline.com).

MultiRATE will rely on the Design Science Research Methodology (DSRM) to validate each of its artefacts. This methodology enables a structured, objective, utility-based, and end-user-centric approach. By conducting design and evaluation activities in iterative cycles, we can elicit frequent feedback and ensure continuous improvement. DRSM incorporates principles, practices, and process models which are adequate to conduct design science research in applied research disciplines, whose cultures value incrementally effective solutions. ^55^ The design science paradigm seeks to create and evaluate “what is effective” in the problem space. ^56^ The design-science paradigm has its roots in engineering and the sciences of the artificial. ^56, 57^ It is fundamentally a problem-solving paradigm. ^56^ More specifically, the process model adopted by MultiRATE is based on the model developed by Peffers, *et al.,* ^58^ presented in Figure 4.

In addition to the DRSM methodology, a standardized certification scheme for individual RL and HRL assessment is proposed to normalize RL assessments across different evaluations and evaluators, ensuring their reliability and consistency. This evaluation scheme establishes formal procedures to harmonize the RL evaluations, ensuring alignment with the defined criteria and methodologies. Additionally, the proposed certification guidelines aim to support future RL assessment schemes that may include various innovation development strategies. The formalized assessment can be used either as a self-assessment or as a third-party assessment practice, depending on the requirements. The standardized guidelines have been tested within civil security projects and stakeholders. ^59^

## Conclusions and discussion

MultiRATE proposes a holistic framework specifically designed to enhance the maturity assessment method for security research and innovation projects from the EU-funded security cluster 3 program. This framework integrates the well-known TRL scale with additional RL scales such as SocRL, SecRL, LPERL, IRL, CRL, and MRL. Additionally, MultiRATE introduces an investment forecasting tool to manage research projects and resources more effectively, relating RL improvement to the required effort. Of course, such a tool is inherently limited by the quality and scope of its training data, as well as the technological variables, and highly related to the context of the specific features required for the forecasting process. This approach aims to address the limitations of the current TRL method, which often fails to fully expose the reasons behind the limited adoption of outcomes from EU-funded security research and innovation projects. MultiRATE’s HRL assessment aims to integrate all these RL dimensions, considering their unique characteristics, to evaluate the readiness of all aspects of a product, system, or process. This integration aims to provide granular insights into progress and feasibility from a holistic perspective. By providing a more comprehensive evaluation from various perspectives, MultiRATE seeks to bridge the “valley of death” between project results and their effective uptake, ultimately enhancing security capabilities within EU member states.

Designing each artefact of the MultiRATE framework involves addressing specific challenges to ensure a comprehensive and effective HRL assessment. For the TRL, the main challenges include developing appropriate indicators for each level, ensuring consistency with existing EU R&D definitions, and accurately assessing the maturity of diverse technologies, including software development. This requires a deep understanding of technological development stages and the ability to create indicators that reflect these stages accurately. The SocRL faces challenges in creating a comprehensive framework that addresses societal readiness across various domains, ensuring societal acceptance and adaptation. This involves understanding societal needs and the factors that influence the adoption of new innovations. The SecRL, which does not have precedents in the literature, must define from scratch security indicators that cover a wide range of threats and measures, ensuring the framework is adaptable to different contexts and technologies. This requires staying updated with emerging security threats and best practices. The LPERL needs to address the complex and evolving legal, privacy, and ethical standards, ensuring the framework is applicable to various technologies and contexts, specifically adapted to the security domain. This involves understanding the legal and ethical implications of new technologies and creating indicators that reflect these considerations. For the IRL, the main challenges include assessing the readiness of interfaces and elements for seamless integration into larger systems, identifying potential risks and dependencies, and ensuring interoperability and documentation standards are met. This requires a thorough understanding of how different components interact and the potential technical issues that may arise during integration. For the CRL, challenges involve evaluating market readiness, managing intellectual property, and developing effective commercialisation strategies. This includes understanding market dynamics, competition, and regulatory requirements, as well as ensuring that the product or technology is ready for market entry and adoption. The MRL faces challenges in ensuring manufacturability, managing supply chain readiness, and addressing quality control and regulatory compliance. This involves assessing the maturity of manufacturing processes, ensuring that production capacity meets demand, and maintaining high standards of quality and efficiency.

On the other hand, for the HRL that integrates all these RL dimensions, the main challenge is how to do it into a cohesive framework, ensuring flexibility and comprehensiveness, and maintaining the unique aspects of each RL dimension. This requires balancing the integration of different domains with the need to preserve their standalone usefulness and designing indicators that allow for a seamless alignment of levels across various RL scales. Additionally, it involves harmonizing the methodologies and indicators from each RL dimension to create a unified assessment framework that provides granular insights into progress and feasibility from a holistic perspective. Finally, for the forecasting module, the challenges lie in predicting funding needs and development progress accurately, standardizing data inputs, and addressing subjectivity in assessments. This involves creating a reliable model that can accurately predict the resources needed for development and the potential progress that can be achieved, while overcoming the variability in data recording across organizations and standardizing the input features.

Future work will focus on implementing these elements by addressing the limitations identified in the TRL framework, ensuring that each readiness level is accurately assessed and aligned with the evolving needs and standards of the European R&D community. DSRM and the network of collaborators can help overcome these challenges by guiding the iterative development and validation of each element, while providing valuable insights and feedback from experts across various fields, ensuring a robust and comprehensive readiness assessment framework.

This paper set out to address a critical gap in the uptake of EU-funded security research by developing MultiRATE, a holistic, multi-dimensional Readiness Level (RL) framework. By integrating seven complementary dimensions—Technology, Societal, Security, Legal/Privacy/Ethical, Integration, Commercialisation, and Manufacturing—the framework goes beyond the traditional TRL to provide a comprehensive maturity assessment. The conceptual design demonstrates that considering these diverse aspects together allows stakeholders to identify hidden bottlenecks, anticipate costs and risks, and align innovation pathways with regulatory, ethical, and societal expectations. From a policy perspective, MultiRATE offers several contributions:

• **Strategic Funding Decisions:** The forecasting module allows policy makers and funding bodies to estimate the effort required to raise maturity across different RLs, enabling more targeted and cost-effective allocation of Horizon Europe and national resources.
• **Evidence-Based Regulation:** The inclusion of Societal (SocRL) and Legal/Privacy/Ethical (LPERL) dimensions provides early insight into public acceptance and compliance issues, supporting the development of harmonized EU regulations.
• **Bridging the “Valley of Death”:** By highlighting integration, commercialisation, and manufacturing readiness alongside technology maturity, the MultiRATE framework helps identify where additional incentives, follow-up funding, or procurement measures are needed to move promising solutions from research to market.
• **Stakeholder Coordination:** The holistic view enables policy makers to coordinate actions among researchers, industry, investors, and end-users, ensuring that maturity improvements in one dimension are not undermined by weaknesses in others.

Looking forward, the MultiRATE methodology can serve as a diagnostic and planning tool for future EU research programmes, not only within civil security but also in other domains where complex socio-technical innovations require cross-dimensional readiness. By making these policy implications explicit, MultiRATE is not only a conceptual advance over TRL but also a practical instrument for shaping European innovation policy, ultimately strengthening the impact of public investments in security research.

## Ethics and consent

Ethical approval and consent were not required.

## Data availability

No data are associated with this article.

## Acknowledgement and disclaimer

Funded by the European Union. Views and opinions expressed are however those of the author(s) only and do not necessarily reflect those of the European Union or REA. Neither the European Union nor the granting authority can be held responsible for them.

## Footnotes

^1^
The nine TRL levels as adopted by the EU are [4][4]: TRL 1 – basic principles observed TRL 2 – technology concept formulated TRL 3 – experimental proof of concept TRL 4 – technology validated in lab TRL 5 – technology validated in relevant environment (industrially relevant environment in the case of key enabling technologies) TRL 6 – technology demonstrated in relevant environment (industrially relevant environment in the case of key enabling technologies) TRL 7 – system prototype demonstration in operational environment TRL 8 – system complete and qualified TRL 9 – actual system proven in operational environment (competitive manufacturing in the case of key enabling technologies; or in space).
