# Supplementary material for: A holistic framework for assessing the uptake potential of EU-funded security research and innovation project results
Source: Open Res Eur. 2026 Apr 29;5:115. Originally published 2025 Apr 28. [Version 3] doi: 10.12688/openreseurope.19711.3 (PMC12949380; doi:10.12688/openreseurope.19711.3)
Supplement: Peer review history

# Reviewer report: Reviewer response for version 3

**DOI:** 10.21956/openreseurope.25672.r73292

**Published:** 2026-05-05

**Authors:** Damien Van Puyvelde

**Affiliations:** 1 Institute of Security and Global Affairs, Leiden University, Leiden, The Netherlands

The authors conducted revisions that addressed my recommendations. I approve of this manuscript.

Where applicable, are recommendations and next steps explained clearly for others to follow? (Please consider whether others in the research community would be able to implement guidelines or recommendations and/or constructively engage in the debate)

Yes

Does the article adequately reference differing views and opinions?

Yes

Are all factual statements correct, and are statements and arguments made adequately supported by citations?

No

Is the rationale for the Open Letter provided in sufficient detail? (Please consider whether existing challenges in the field are outlined clearly and whether the purpose of the letter is explained)

Yes

Is the Open Letter written in accessible language? (Please consider whether all subject-specific terms, concepts and abbreviations are explained)

Yes

Reviewer Expertise:

Security studies, Governance, Emerging technologies

I confirm that I have read this submission and believe that I have an appropriate level of expertise to confirm that it is of an acceptable scientific standard.

# Reviewer report: Reviewer response for version 2

**DOI:** 10.21956/openreseurope.24091.r67974

**Published:** 2026-03-04

**Authors:** Julia Bachtrögler Unger

**Affiliations:** 1 Austrian Institute of Economic Research, Vienna, Austria

I am fine with the revised article

Where applicable, are recommendations and next steps explained clearly for others to follow? (Please consider whether others in the research community would be able to implement guidelines or recommendations and/or constructively engage in the debate)

Yes

Does the article adequately reference differing views and opinions?

Partly

Are all factual statements correct, and are statements and arguments made adequately supported by citations?

Yes

Is the rationale for the Open Letter provided in sufficient detail? (Please consider whether existing challenges in the field are outlined clearly and whether the purpose of the letter is explained)

Partly

Is the Open Letter written in accessible language? (Please consider whether all subject-specific terms, concepts and abbreviations are explained)

Yes

Reviewer Expertise:

Regional economics; Evaluation of EU cohesion policy, with a focus on research and innovation projects (including synergies with Horizon 2020).

I confirm that I have read this submission and believe that I have an appropriate level of expertise to confirm that it is of an acceptable scientific standard.

# Reviewer report: Reviewer response for version 2

**DOI:** 10.21956/openreseurope.24091.r69302

**Published:** 2026-02-27

**Authors:** Mary Goldberg

**Affiliations:** 1 University of Pittsburgh, Pittsburgh, Pennsylvania, USA

Several updates have been made to the manuscript in response to reviewer feedback. Statements have been added to include conceptual framework rather than presenting empirical results or finalized metrics, intended audiences, standalone methods, research questions, and study key contributions and policy implications. I believe the article is suitable for indexing given these updates.

Where applicable, are recommendations and next steps explained clearly for others to follow? (Please consider whether others in the research community would be able to implement guidelines or recommendations and/or constructively engage in the debate)

Yes

Does the article adequately reference differing views and opinions?

Yes

Are all factual statements correct, and are statements and arguments made adequately supported by citations?

Yes

Is the rationale for the Open Letter provided in sufficient detail? (Please consider whether existing challenges in the field are outlined clearly and whether the purpose of the letter is explained)

Yes

Is the Open Letter written in accessible language? (Please consider whether all subject-specific terms, concepts and abbreviations are explained)

Yes

Reviewer Expertise:

education, technology transfer, assistive technology

I confirm that I have read this submission and believe that I have an appropriate level of expertise to confirm that it is of an acceptable scientific standard.

# Reviewer report: Reviewer response for version 2

**DOI:** 10.21956/openreseurope.24091.r68273

**Published:** 2026-02-13

**Authors:** Damien Van Puyvelde

**Affiliations:** 1 Institute of Security and Global Affairs, Leiden University, Leiden, The Netherlands

This is an interesting project/letter that provides a comprehensive summary of key issues with TRL, especially in this specific context. The authors propose an ambitious way forward to mitigate the issues they identify. The paper makes well-taken points that are likely to be relevant to a large audience of researchers and practitioners. I only have two comments.

 (More important) The methodology section (p.5) requires additional detail to improve transparency and reproducibility. The authors should clarify how the literature review was conducted (e.g. databases consulted, timeframe, inclusion/exclusion criteria, and approach to synthesis). Further explanation is needed regarding the selection, assessment, and modification of the seven readiness level frameworks, as well as how the work conducted in WP2 of the MultiRATE project informed the analysis presented in this paper.

 (Minor) Page 4: How do you know that the uptake problem is mostly due to a "fragmentation of the market". This might require more careful phrasing. Is this a fact or a hypothesis, either way, where does it come from? Aren't there several other possible hypotheses, as the rest of the paper suggests (e.g. issues with demand side, supply side, institutional processes, design, etc.) ?

Where applicable, are recommendations and next steps explained clearly for others to follow? (Please consider whether others in the research community would be able to implement guidelines or recommendations and/or constructively engage in the debate)

Yes

Does the article adequately reference differing views and opinions?

Yes

Are all factual statements correct, and are statements and arguments made adequately supported by citations?

No

Is the rationale for the Open Letter provided in sufficient detail? (Please consider whether existing challenges in the field are outlined clearly and whether the purpose of the letter is explained)

Yes

Is the Open Letter written in accessible language? (Please consider whether all subject-specific terms, concepts and abbreviations are explained)

Yes

Reviewer Expertise:

Security studies, Governance, Emerging technologies

I confirm that I have read this submission and believe that I have an appropriate level of expertise to confirm that it is of an acceptable scientific standard, however I have significant reservations, as outlined above.

# Response

**Published:** 2026-04-16

**Authors:** Luis Unzueta

Thank you for the valuable remarks. The literature review conducted in this paper follows a narrative approach. It aimed at identifying key concepts, limitations of existing frameworks, and factors that influence the uptake of EU-funded security research. Based on these, the purpose of this paper was more conceptual rather than systematic or empirical, so no formal systematic review protocol (e.g., PRISMA) was applied. We have clarified this in the "Methodology" section of the manuscript's new version. On the other hand, we agree that the statement regarding market fragmentation required more careful phrasing. Our intention was not to present fragmentation as the sole or dominant cause, but rather as one of several factors identified in the literature and policy reports. We have clarified this in the "Introduction" section of the manuscript's new version.

# Reviewer report: Reviewer response for version 1

**DOI:** 10.21956/openreseurope.21321.r55191

**Published:** 2025-08-29

**Authors:** Andrea Gatto

**Affiliations:** 1 Wenzhou-Kean University, Wenzhou, China

The submission at hand deals with EU-funded security research and innovation. The paper is well drafted and conveniently organised. The article is clear and is corroborated by several diagrams. 

 - The topic may be better introduced, referring to the latest scholarly and policy literature.

 - The authors shall further clarify their research design, research goals, and, above all, research question throughout the text. Likewise, the policy relevance should emerge more clearly.

 - The paper should be better framed within the current policy scenario and related literature. I suggest reviewing additional sector publications and exploring related subjects more attentively. The authors may want to check recent European Commission, EU and policy papers, as well as scholarly publications in field journals.

 - Many statements and sentences would be better corroborated by additional citations; grey literature is fine to a limited extent, I recommend adding scientific publications as well.

 - The methodology section should be expanded and include more details about the techniques used and the steps undertaken.

 - The conclusion and policy implications part is currently dry. I recommend extending it to better wrap up the study's findings and better sell the paper.

Where applicable, are recommendations and next steps explained clearly for others to follow? (Please consider whether others in the research community would be able to implement guidelines or recommendations and/or constructively engage in the debate)

Partly

Does the article adequately reference differing views and opinions?

Partly

Are all factual statements correct, and are statements and arguments made adequately supported by citations?

Partly

Is the rationale for the Open Letter provided in sufficient detail? (Please consider whether existing challenges in the field are outlined clearly and whether the purpose of the letter is explained)

Partly

Is the Open Letter written in accessible language? (Please consider whether all subject-specific terms, concepts and abbreviations are explained)

Yes

Reviewer Expertise:

Economics; economic policy; environmental policy; innovation policy

I confirm that I have read this submission and believe that I have an appropriate level of expertise to confirm that it is of an acceptable scientific standard, however I have significant reservations, as outlined above.

# Response

**Published:** 2025-12-17

**Authors:** Luis Unzueta

*Comment 2.1: The submission at hand deals with EU-funded security research and innovation. The paper is well drafted and conveniently organised. The article is clear and is corroborated by several diagrams.* *- The topic may be better introduced, referring to the latest scholarly and policy literature.*

**Response: **In the Introduction section, the wordings that describe the goal of the article is now improved. Furthermore, a Methodology section is added and in the sections that describe the various RLs (Readiness Levels), additional literature references have been added.

*Comment 2.2: The authors shall further clarify their research design, research goals, and, above all, research question throughout the text. Likewise, the policy relevance should emerge more clearly.*

**Response: **We revised the manuscript to state the research question more explicitly (Abstract section)—namely, how a holistic multi-dimensional readiness framework can overcome the limitations of the TRL and improve the uptake of EU-funded security research results. We also clarified our research design (a conceptual, design-science approach with iterative development and validation) and research goals (to define and integrate seven RL dimensions and a forecasting module for maturity assessment) (Introduction and Methodology section). In addition, we strengthened the means of the framework’s policy relevance, highlighting its potential to guide EU funding decisions, support strategic investment, and inform policy measures that foster innovation uptake.

*Comment 2.3: The paper should be better framed within the current policy scenario and related literature. I suggest reviewing additional sector publications and exploring related subjects more attentively. The authors may want to check recent European Commission, EU and policy papers, as well as scholarly publications in field journals.*

**Response: ** In the sections that describe the various RLs (Readiness Levels), additional literature references have been added.

*Comment 2.4: Many statements and sentences would be better corroborated by additional citations; grey literature is fine to a limited extent, I recommend adding scientific publications as well.*

**Response: **In the sections that describe the various RLs (Readiness Levels), additional literature references have been added.

*Comment 2.5: The methodology section should be expanded and include more details about the techniques used and the steps undertaken.*

**Response: **A separate methodology section has been added to the article to clarify the followed methodology.

 “ **Methodology:** The factors that contribute to success and failure of the uptake of EU-funded security cluster 3 program projects (Civil Security for Society) and the limitations of TRL are collected via a narrative literature review  that was started with a search on main keyword (‘’TRl shortcomings’’, ‘’Factors uptake EU Research’’, ‘’Evaluation EU funded research’’) followed by citation research (snowballing approach). For the purpose of assessing the maturity of EU R&D project outputs in the security domain, seven existing readiness levels (RL) were studied, assessed, and modified. These two steps reflect part of the work done in WP2 of the EU-MultiRATE project.”

*Comment 2.6: The conclusion and policy implications part is currently dry. I recommend extending it to better wrap up the study's findings and better sell the paper.*

**Response: **The text in Conclusion section has been expanded and restructured to better summarise the paper’s key contributions and to emphasise the policy implications of the MultiRATE framework. It now outlines its potential to support strategic EU funding decisions, anticipate societal and legal challenges, and bridge the “valley of death” between research results and market adoption.

# Reviewer report: Reviewer response for version 1

**DOI:** 10.21956/openreseurope.21321.r55189

**Published:** 2025-08-21

**Authors:** Julia Bachtrögler Unger

**Affiliations:** 1 Austrian Institute of Economic Research, Vienna, Austria

**Summary:**

 The Open Letter outlines the conceptual framework developed by the MultiRATE EU research project, which aims to complement the traditional Technological Readiness Level (TRL) metric with a multi-dimensional readiness assessment. The authors argue that reliance on TRL alone cannot adequately explain the persistent gap between research results and market adoption in innovations in the field of security. They propose the introduction of additional readiness dimensions - such as Societal, Legal, Commercialization or Manufacturability - to reflect the multifaceted maturity of research outputs. The Open Letter primarily serves as a forward-looking conceptual contribution, outlining principles and intentions rather than presenting final results or validated tools.

 The motivation and relevance of the work are well presented. However, to help set expectations appropriately for readers, I recommend that the authors clarify at the outset that this Open Letter serves as a conceptual roadmap rather than presenting empirical outcomes or finalized metrics. Additionally, to support broader comprehension, especially for audiences less familiar with TRLs, I suggest adding a list of the conventional TRL stages in a footnote.

 All in all, this Open Letter offers an interesting and relevant contribution to the debate on the uptake of EU-funded (security) research and innovation project results. I support its indexing, with the recommendation to elaborate further on the target users and implementation challenges (see my comments below). 

**More detailed comments on the multi-dimensional assessment of technological readiness:**

 The proposed expansion of the assessment of technological readiness to include multiple RL dimensions is intuitive and addresses the limitations of the current measurement scheme when it comes to explaining the “the Death Valley” between research output (opportunity discovery) and adoption (product development).

 Nevertheless, several challenges should be acknowledged more explicitly. The operationalization of each RL dimension will inevitably involve high complexity due to heterogeneity in technologies and implementation contexts (such as legal frameworks) across the EU. For example, the Legal, Privacy and Ethical RLs are context-specific and likely to vary significantly between countries or even regions. Similarly, the Integration RL appears particularly difficult to harmonize across technological domains and sectors. While the Holistic RL dimension is introduced, its added value relative to the individual RL dimensions remains unclear; I would encourage the authors to further justify its inclusion.

 It is not totally clear, which stakeholder group(s) should be addressed with the multi-dimensional RL approach or whether target groups addressed vary by RL dimension. Clarifying the intended audience for each dimension and whether the framework aims to support policy-level evaluations, serve as a diagnostic tool for analyzing systemic barriers, or guide practitioners involved in specific stages in specific projects (e.g., at the frontier between R&D and adoption, or investors) would improve the usability and applicability of the approach.  

 Finally, the investment forecasting module appears highly promising and could become a valuable resource for technology developers and investors. However, also here, its feasibility and robustness will likely depend heavily on the technological and contextual variables in my view.

Where applicable, are recommendations and next steps explained clearly for others to follow? (Please consider whether others in the research community would be able to implement guidelines or recommendations and/or constructively engage in the debate)

Yes

Does the article adequately reference differing views and opinions?

Partly

Are all factual statements correct, and are statements and arguments made adequately supported by citations?

Yes

Is the rationale for the Open Letter provided in sufficient detail? (Please consider whether existing challenges in the field are outlined clearly and whether the purpose of the letter is explained)

Partly

Is the Open Letter written in accessible language? (Please consider whether all subject-specific terms, concepts and abbreviations are explained)

Yes

Reviewer Expertise:

Regional economics; Evaluation of EU cohesion policy, with a focus on research and innovation projects (including synergies with Horizon 2020).

I confirm that I have read this submission and believe that I have an appropriate level of expertise to confirm that it is of an acceptable scientific standard, however I have significant reservations, as outlined above.

# Response

**Published:** 2025-12-17

**Authors:** Luis Unzueta

*Comment 1.1: The Open Letter outlines the conceptual framework developed by the MultiRATE EU research project, which aims to complement the traditional Technological Readiness Level (TRL) metric with a multi-dimensional readiness assessment. The authors argue that reliance on TRL alone cannot adequately explain the persistent gap between research results and market adoption in innovations in the field of security. They propose the introduction of additional readiness dimensions - such as Societal, Legal, Commercialization or Manufacturability - to reflect the multifaceted maturity of research outputs. The Open Letter primarily serves as a forward-looking conceptual contribution, outlining principles and intentions rather than presenting final results or validated tools.* *The motivation and relevance of the work are well presented. However, to help set expectations appropriately for readers, I recommend that the authors clarify at the outset that this Open Letter serves as a conceptual roadmap rather than presenting empirical outcomes or finalized metrics. Additionally, to support broader comprehension, especially for audiences less familiar with TRLs, I suggest adding a list of the conventional TRL stages in a footnote.* *All in all, this Open Letter offers an interesting and relevant contribution to the debate on the uptake of EU-funded (security) research and innovation project results. I support its indexing, with the recommendation to elaborate further on the target users and implementation challenges (see my comments below).*

 Based on this comment, a sentence has been added to the article to make clear the RLs are described at a conceptual level. “This open letter is meant to describe at a conceptual level how a successor of the TRL metric could look like. We will not present empirical outcomes nor finalised metrics.’’ Besides, the conventional TRL stages in the form as adopted by the EU have been added in a footnote.

*Comment 1.2: The proposed expansion of the assessment of technological readiness to include multiple RL dimensions is intuitive and addresses the limitations of the current measurement scheme when it comes to explaining the “the Death Valley” between research output (opportunity discovery) and adoption (product development).* *Nevertheless, several challenges should be acknowledged more explicitly. The operationalization of each RL dimension will inevitably involve high complexity due to heterogeneity in technologies and implementation contexts (such as legal frameworks) across the EU. For example, the Legal, Privacy and Ethical RLs are context-specific and likely to vary significantly between countries or even regions. Similarly, the Integration RL appears particularly difficult to harmonize across technological domains and sectors. While the Holistic RL dimension is introduced, its added value relative to the individual RL dimensions remains unclear; I would encourage the authors to further justify its inclusion.*

 Using the individual RL dimensions, like the presented SocRL, SecRL, LPERL, IRL, CRL, MRL, or any other available in the related literature, apart from TRL, can give us a better understanding of a system’s overall maturity than using TRL alone. However, these RL dimensions can be quite heterogeneous (i.e., different number of levels, different relations between indicators and levels, etc.) and their alignment might not be clear enough (e.g., interrelations among indicators, groups of indicators, levels, etc.) without a proper harmonization among them. This can add some difficulties for a proper holistic maturity assessment. As explained in the paper, different strategies have been proposed in the literature to integrate different RL dimensions for a holistic assessment, some more flexible and some other less flexible with respect to the design of the individual RL dimensions. We think that having flexibility in the design of each RL dimension is preferable so that we can keep the standalone usefulness. However, we still need some common ground for their integration and for that purpose we believe that using a reference HRL dimension could be beneficial. For instance, the levels of this HRL could be aligned to some indicators, group of indicators, levels of each RL dimension and, at the same time, to some specific events related to holistic maturity progress (e.g., having accomplished some technological, societal, legal, commercial, etc. achievement). Besides, once the RL dimensions alignment is achieved with the support of this HRL, the holistic assessment score obtained by this HRL can also give us a quick understanding of the achieved holistic maturity that can be complemented with a more detailed review of the scores obtained in each RL dimension. Based on this comment, we have updated the section about HRL with these explanations.

*Comment 1.3: It is not totally clear, which stakeholder group(s) should be addressed with the multi-dimensional RL approach or whether target groups addressed vary by RL dimension. Clarifying the intended audience for each dimension and whether the framework aims to support policy-level evaluations, serve as a diagnostic tool for analyzing systemic barriers, or guide practitioners involved in specific stages in specific projects (e.g., at the frontier between R&D and adoption, or investors) would improve the usability and applicability of the approach.*

 The MultiRATE framework targets a broad set of stakeholders, but each RL dimension has primary audiences. TRL and MRL mainly serve R&D teams, industry, and investors; SocRL and LPERL inform policy makers and societal/ethical experts; SecRL and IRL support practitioners and integrators; and CRL aids commercialisation teams and investors. The holistic RL provides a cross-dimensional view for policy evaluation, investment decisions, and diagnosing systemic barriers.

*Comment 1.4: Finally, the investment forecasting module appears highly promising and could become a valuable resource for technology developers and investors. However, also here, its feasibility and robustness will likely depend heavily on the technological and contextual variables in my view.*

 The reviewer is quite correct in pointing out that the module's performance relies on technological and contextual variables. This is, of course, a foundational principle in forecasting. Our work already incorporates state-of-the-art mechanisms to model and adapt to these dependencies, allowing it to be as objective as possible given the nature of the variables used. The reviewer’s comment has been addressed in Conclusions’ section.
